# Grazing-incidence small-angle neutron scattering from structures below an interface

**DOI:** 10.1107/S1600576717007518

**Published:** 2017-07-07

**Authors:** Shirin Nouhi, Maja S. Hellsing, Vassilios Kapaklis, Adrian R. Rennie

**Affiliations:** a Division for Materials Physics, Department of Physics and Astronomy, Uppsala University, Box 516, 751 20, Uppsala, Sweden

**Keywords:** grazing-incidence small-angle neutron scattering, GISANS, colloidal particles, solid/liquid interfaces

## Abstract

Changes of scattering are observed as the grazing angle of incidence of an incoming beam increases and probes different depths in samples. A model has been developed to describe the observed intensity in grazing-incidence small-angle neutron scattering (GISANS) experiments. This includes the significant effects of instrument resolution, the sample transmission, which depends on both absorption and scattering, and the sample structure. The calculations are tested with self-organized structures of two colloidal samples with different size particles that were measured on two different instruments. The model allows calculations for various instruments with defined resolution and can be used to design future improved experiments. The possibilities and limits of GISANS for different studies are discussed using the model calculations.

## Abstract 2

Calculations of intensity in grazing-incidence small-angle neutron scattering are made for colloidal structures near a solid/liquid interface.

## 1. Introduction   

The special role of interfaces is crucial in many applications of materials and in physical and chemical processes that include catalysis, lubrication and corrosion (Somorjai & Li, 2011 ▸; Allara, 2005 ▸). Similarly in subjects such as biological and environmental science, interactions at boundaries are important (Nel *et al.*, 2009 ▸; Höök *et al.*, 2008 ▸). Surface and interface science has attracted considerable attention in recent decades, and with increasing interest, many surface-sensitive experimental methods have been developed, such as scanning force microscopy (Karoutsos, 2009 ▸), electron microscopy (Kimoto *et al.*, 2007 ▸) and fluorescence microscopy (Harootunian *et al.*, 1986 ▸), as well as X-ray photo-electron spectroscopy (Ruta *et al.*, 2012 ▸), surface plasmon spectroscopy (Mulvaney, 1996 ▸), and other surface-sensitive X-ray (Wiegart *et al.*, 2009 ▸) and neutron scattering techniques (Penfold & Thomas, 2014 ▸). Understanding the advantages and limitations of each technique is important in order to obtain information about a specific structure.

Neutron and X-ray scattering experiments have several features in common, and in contrast to microscopy, which produces images by means of reconstruction with lenses or by scanning probes, they provide averaged statistical information from large areas or volumes of the sample (Squires, 2009 ▸; Pershan & Schlossman, 2012 ▸; Sivia, 2011 ▸). Neutron scattering benefits from contrast variation using isotopic labelling and has higher penetration through many materials compared to other techniques. This makes neutrons a powerful tool to determine structures at buried interfaces such as those between solids and liquids. Calculations are needed to interpret data quantitatively. The scale of structural features such as the spacing of particles is often readily identified from the position of peaks in momentum transfer space in scattering patterns. Interpreting the degree of correlation between objects and details of structural arrangements usually relies on understanding the observed intensity distribution and relating this to model structures. The calculation of the scattering will also depend on the penetration of the radiation to different depths, and in this respect there are useful similarities of neutron studies with various near-surface spectroscopy methods such as infrared attenuated total reflection and fluorescence techniques, as well as scattering experiments with both visible light and X-rays (Axelrod *et al.*, 1983 ▸; Fish, 2001 ▸; Rivers *et al.*, 1991 ▸).

In recent decades, specular neutron reflection has developed rapidly as a valuable tool to study the structure of nanoscale films of hard and soft matter by providing information about the density profile in the direction of the interface normal. Additionally, the in-plane structure of the interface can be studied when illuminating the sample at low angles that are close to the critical angle, by observing the scattering away from the specular direction.

We have previously shown that colloidal particles can self-assemble and form large domains of oriented and regular structure near solid/liquid interfaces (Hellsing, Kapaklis *et al.*, 2012 ▸). These systems have potential applications as, for example, templates for patterning to create photonic devices (Kosiorek *et al.*, 2004 ▸). Studying self-organizing systems is a means to understand particle/particle and particle/surface interactions, packing, ordering, and dynamic behaviour of crystalline systems (Ottewill, 1989 ▸; Pusey *et al.*, 1989 ▸).

In this article we describe the use of grazing-incidence small-angle neutron scattering (GISANS) to study structures of colloidal dispersions that self-assemble at interfaces into crystalline structures with ordered and aligned regions extending several micrometres from the solid/liquid interface. The penetration depth of the beam and scattering signal are modelled using known properties of the materials and with allowance for the effects of a spread of wavelength and the angular divergence of the beam. This model will be compared with new experimental data for a colloidal dispersion of latex particles at a solid/liquid interface. Discussions on the accessibility and limitations of the GISANS technique to determine such structures will follow.

### 1.1. Theory   

**Figure 1:**
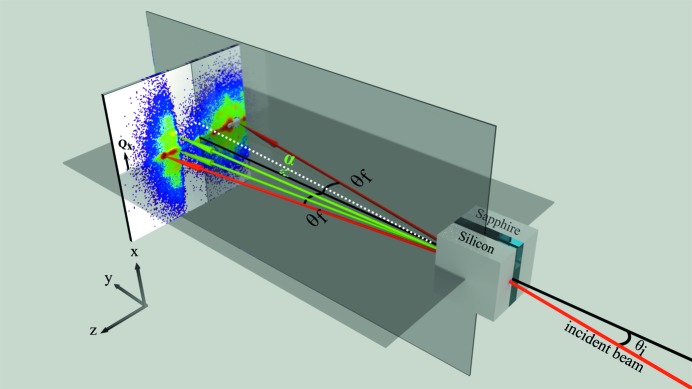
Schematic geometry of the GISANS experiment with a vertical sample holder (D22 and NG3-SANS). The dispersion is sealed between the silicon and sapphire crystals. 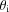 is the incident angle of the beam; 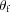 is the angle of the scattered beam on the horizontal plane; α is the angle of the scattered beam on the vertical plane. A scattered beam with 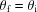 is shown with red arrows and a scattered beam with 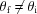 in green.

In GISANS experiments, the beam is tightly collimated so as to provide good angular resolution. In order to determine the structure of dispersions at solid/liquid interfaces, a sample geometry is chosen with the incident beam entering the edge face of a crystal substrate. It then impinges on the solid/liquid interface at small angles close to the critical angle for total reflection, where it is reflected and scattered. Fig. 1 ▸ shows a GISANS experiment setup with vertical sample geometry. At incident angles below the critical angle for total reflection, only an evanescent wave extends into the liquid phase and the penetration depth is determined by the interface ‘contrast’, that is the difference in refractive index for neutrons of the two bulk phases. Above the critical angle, a refracted beam enters the liquid and the penetration is limited generally both by the absorption of the beam by the sample and by the various scattering processes. There are a number of physical processes that can play important roles in these types of experiments that are less common in other scattering studies, and the circumstances in which these can be important are mentioned briefly in the following paragraphs.

The decay of the amplitude of the wave due to absorption and scattering is characterized by an attenuation coefficient (μ). The low angles and large sample lengths give rise to long paths through the materials and so the attenuation is often quite large. In the case of grazing-incidence small-angle X-ray scattering (GISAXS), the absorption is normally dominant. For neutrons, the absorption is often smaller and the scattering (mostly incoherent scattering that is strong when the sample contains H_2_O or other hydrogenous material) is the dominant cause of attenuation, which gives an effect analogous to absorption in reducing the neutron beam. The characteristic decay length of the wave in GISANS experiments is called the penetration depth and is defined as the distance at which the wave amplitude drops to (1/*e*) of the value at the interface. By careful choice of the materials forming the interface, the wavelength and the incident angle, it is possible to select the average depth of penetration of the beam in the sample and thus control the volume or mean depth of the sample that contributes to the measured scattering signal. This allows measurements of the structure at different depths. Knowledge of the wave amplitude is also required to calculate the scattering, which is obtained from the square of the sum of the amplitudes for the coherent scattering. The long path length of the beam in the sample has a further consequence when there is scattering, as the probability of multiple scattering can be significant.

In simple scattering theory, it is usually assumed that one can add the scattering from each nucleus in the sample in order to calculate the total scattering. This approach, which treats the perturbation of the wave as weak, is known as the Born approximation. This is also only valid if a neutron is scattered once passing through the sample. In the case of grazing-incidence experiments, where neutrons are totally reflected from the surface, there is no longer weak interaction so the Born approximation is not adequate. The distorted wave Born approximation (DWBA) is an extension to the calculation that considers the perturbation of the incident wave and has been applied to calculate diffuse scattering, such as that from rough interfaces (Sinha *et al.*, 1988 ▸; Dietrich & Haase, 1995 ▸). Recently, packages for these calculations such as *BornAgain* (Durniak *et al.*, 2015 ▸) and *IsGISAXS* (Lazzari, 2002 ▸) have been developed in a way that scattering can be treated with the DWBA approach for various structures at different interfaces, particularly those of solids and air or vacuum. There are also algorithms treating samples as layers, such as the multi-slice DWBA approach, which has been used to calculate X-ray scattering at grazing incidence (Venkatakrishnan *et al.*, 2016 ▸). When measuring solid/air interfaces, it is often known where the structure has formed, so that other parameters are adjusted in order to measure the scattering pattern from that range of depth. In systems where the structure can form with an unknown separation from the interface, the experiment becomes more depth sensitive. Probing this spacing as well as the structure becomes a great challenge.

The penetration depth has been calculated in several different ways in the literature and various different expressions have been presented, which can be confusing both as regards the different notation that is used and the different approximations that are assumed. In the majority of the studies (Müller-Buschbaum *et al.*, 2008 ▸; Als-Nielsen *et al.*, 1994 ▸; Prieve & Walz, 1993 ▸; Dosch *et al.*, 1986 ▸; Wolff *et al.*, 2014 ▸; Al-Hussein *et al.*, 2013 ▸; Parratt, 1954 ▸; Dietrich & Wagner, 1984 ▸; Gutfreund, 2011 ▸), the penetration depth can be expressed as
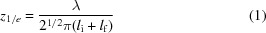
with 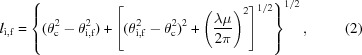
where 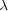 is the wavelength, 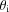 the incident beam angle, 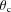 the critical angle for the given interface and wavelength, and 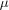 the attenuation coefficient. According to Snell’s law the critical angle is given by 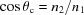, where *n* is the refractive index for neutrons of each material and is given by 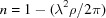. The equation can be simplified so that the scattering angle, 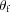, is equal to 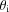, and then the penetration depth of the beam varies only with respect to the incident beam:
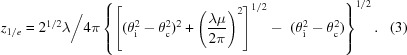
For X-rays 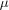 is usually taken as the absorption coefficient.

Some reports (*e.g.* Frielinghaus *et al.*, 2012 ▸; Kerscher *et al.*, 2011 ▸) describe the penetration depth only below the critical angle. Their expression gives a value for 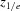 that is twice that calculated by equation (3) and would correspond to the decay of intensity rather than amplitude, which is not expressly stated in those papers.

The penetration depth has been introduced as a straightforward description of the depth sensitivity of the GISANS and GISAXS techniques in various papers. However, as we will show, depending on the resolution of the instrument, the scattering signal from the sample beneath the interface can appear even when the mean angle of incidence is below the critical angle because of the spread of wavelength and the angular divergence. In systems where accurate investigation of structure at different depths in the sample is important, the penetration must be modelled more precisely.

The magnitude of the attenuation coefficient can be assessed from the known properties of materials for neutrons and X-rays: 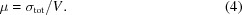
Here, 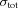 is the total cross section and is given as the sum over various wavelength-dependent components of the cross section for a volume *V*: 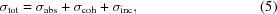
and the three terms correspond to the absorption, the coherent scattering cross section and the spin incoherent scattering cross section, respectively. For X-rays the last term is not relevant. For neutron experiments with a wide range of wavelengths such as with time-of-flight white-beam instruments, the calculations must take into account the wavelength dependence of the absorption and scattering.

A further factor that needs general consideration is the effect of refraction on distorting the scattering, which can be significant in the case of near-small-angle surface scattering (as described by Hamilton *et al.*, 1996 ▸). The refraction of the scattered neutrons, as the angle with the interface is different from that of the incident beam, is not simply an inversion of the effect that occurs with the incident beam. For the case of neutron instruments the wavelength spread is often significant and as the refraction is wavelength dependent this will give rise to a spatial spread of the beam that is correlated with wavelength. These effects are particularly large near the critical angle.

## 2. Samples and measurements   

### 2.1. Materials   

Colloidal dispersions of charge-stabilized polystyrene latices, PS3 and PS11, dispersed in D_2_O have been used for the experiments in this study. The particles are spherical and monodisperse and have been characterized using several methods including zeta potential, scanning electron microscopy, atomic force microscopy, light scattering, small-angle X-ray and neutron scattering *etc.* (Hellsing, Kapaklis *et al.*, 2012 ▸; Hellsing, Rennie *et al.*, 2012 ▸).

The PS3 latex particles have 72 nm radius (polydispersity <1%), about −30 mV surface potential and a density of 1.05 g cm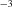. The concentration of the sample was measured to be 9 wt% by drying to constant weight.

The PS11 latex particles have 35 nm radius (polydispersity <5%) and about −35 mV surface potential with a density of 1.05 g cm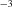. The concentration of the sample was 7 wt%.

SANS and GISANS measurements show several orders of Bragg diffraction peaks, suggesting that particles dispersed in water order to a depth of at least 1 µm into the bulk, forming a face-centred cubic structure with the (111) plane parallel to the solid surface (Hellsing, Kapaklis *et al.*, 2012 ▸; Rennie *et al.*, 2013 ▸; Hellsing, Rennie *et al.*, 2012 ▸).

A study using a quartz crystal microbalance with dissipation (QCM-D) for various different particle and salt concentrations indicated that the particles are close to the interface but not bound at the solid surface (Hellsing & Höök, 2017 ▸).

## 3. Measurements   

In the present experiments, 50 × 50 × 10 mm sapphire (0001) and silicon (111) crystals were used as the solid surfaces. The crystals were cleaned with dilute detergents in H_2_O for a few minutes and then rinsed repeatedly with pure water. The cleaning was continued with sulfuric acid and water (as described in the supporting information) until the surfaces became hydrophilic with no measurable contact angle for water. A sample holder was used (Rennie *et al.*, 2015 ▸), where the sample was contained by a 2 mm thick polytetrafluoro­ethylene gasket between the two crystals. One side was silicon, which provides sufficient contrast with D_2_O for the GISANS experiment, and the other side a transparent sapphire crystal, which allows checking the condition of the sample. The combined influence of solid/liquid and liquid/air interfaces with Poiseuille flow can influence the ordering of particles. Sample handling was performed as reported in our previous paper (Hellsing, Kapaklis *et al.*, 2012 ▸). It has been observed experimentally that gentle flow of the air/latex meniscus across the crystal surface can advance ordering of the particles. The sample was injected at ∼1 ml min^−1^ (average Peclet number <10^−3^) into the empty cell when the cell was rotated 45° with the filling port at the bottom.

The sample cell was placed with the solid/liquid interface vertical on the sample stage as shown in Fig. 1 ▸. The *z* axis is defined perpendicular to the interface, with the positive direction increasing away from the reflecting surface. An increase in depth into the sample is represented by larger negative *z* values. Following the conventional right-hand rule in this geometry, the *y* axis is the direction of the beam and the *x* axis is in the vertical direction. Consequently for all incident angles, *Q* 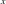 will be mapped on to the vertical axis of the detector and 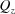 on the horizontal axis.

Measurements on PS3 latex were performed on the D22 instrument at the Institut Laue–Langevin (ILL), Grenoble, with a source to sample distance of 17.6 m and source aperture 20 mm (circular). With a wavelength of 1.4 nm, sample to detector distance of 12 m and slit size of 0.3 mm, the footprint of the beam was 38 × 38 mm at the critical angle and sufficiently high resolution could be obtained for the experiment (https://www.ill.eu/instruments-support/instruments-groups/). The wavelength spread of the incident direct beam was measured on D22 using a small chopper and time-of-flight mode with the collimation used for the scattering experiment and is shown to have a triangular distribution with 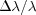 (FWHM) = 0.097 (Fig. S1 supplementary document). The measurements on PS11 that are shown were performed on the NG3 SANS (Glinka *et al.*, 1998 ▸) instrument at the National Institute of Standards and Technology (NIST) Center for Neutron Research (NCNR). The wavelength was set to 0.8 nm, with a source aperture of 14.4 mm, a source to sample distance of 15.7 m was chosen, and the sample to detector distance was set to 13.1 m. The wavelength spread was measured separately and shown to have a triangular distribution with FWHM = 0.124 (Fig. S2 supplementary document).

## 4. Results and discussions   

**Figure 2:**
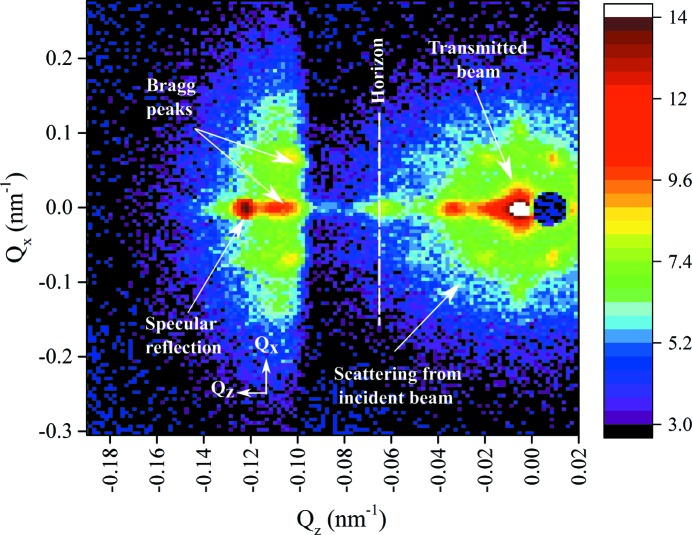
Detector image recorded for PS11 latex in D_2_O at 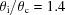, indicating scattering regions (NG3 SANS, NIST). The critical angle for this sample, measured with λ = 0.8 nm, was 0.7°. Streaking of the scattering around the labelled Bragg peaks shows the effect of instrument resolution. This effect is more pronounced with NG3 SANS than D22 owing to the slightly larger wavelength spread and better pixel resolution. The region around the transmitted beam shows the refraction and the scattered signal with clear diffraction peaks from a highly ordered sample structure, but these are subject to heavy multiple scattering. There is also strong diffuse background scattering from the sample cell and sealing ring in this region. In general, describing *Q* is complicated because of both refraction effects and the different origins of the scattering. The axes are labelled simply according to equations (6) and (8). The intensities are normalized and shown on a 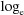 scale.

Fig. 2 ▸ shows an example of the detector image for the PS11 latex dispersed in D_2_O measured on the NG3 instrument. The different scattering regions and the axes, which are similar to those for the measurements of PS3 on D22, are marked on the figure.

**Figure 3:**
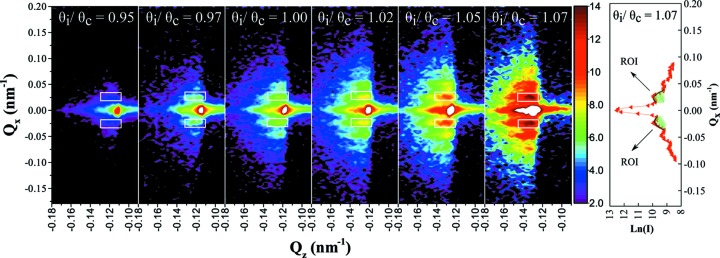
Scattering intensities recorded close to the critical angle from 9 wt% PS3 latex in D_2_O against a silicon surface with D22. Density measurements showed that the particles were dispersed in a 15:85 mixture of H_2_O:D_2_O. Using the scattering length density of this combination the critical angle measured with λ = 1.4 nm was 0.8°. The rectangles around the first-order Bragg peaks represent the regions of interest chosen to estimate the relative intensities plotted in Fig. 5. The intensities are normalized to the measurement time and shown on a 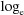 scale. The vertical strip along the central part of 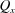 on the right side of the figure shows the integrated peaks as an example for 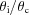 = 1.07.

The conversion to 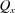 and 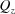 at each measured angle is made using the following expressions:
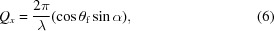


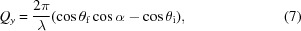


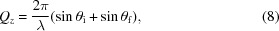
where 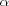 is the angle between the projection of the scattered beam on the vertical plane and the direct beam. It is important to note that each detector image contains some three-dimensional information, but as 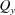 is small the data are essentially two dimensional in 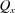 and 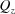 space; however, the signal with different 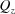 values also contains information from different 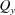. Fig. 3 ▸ shows the scattering pattern at several selected angles close to the critical angle recorded for the PS3 latex at the silicon/D_2_O interface at the D22 instrument. Similar scattering patterns (different *Q* values) were recorded for PS11 with NG3 SANS.

Several orders of Bragg peaks which are equally spaced in 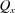 are an indication of a highly ordered sample. The Bragg peak positions correspond to the *d* spacing of the (111) planes (with an uncertainty of 15% due to the detector pixel resolution) for a face-centred cubic structure, which are aligned parallel with the interface, as observed in a previous study (Hellsing, Kapaklis *et al.*, 2012 ▸).

For a single wavelength and no angular dispersion, below the critical angle, the evanescent wave can penetrate a few nanometres into this sample, which is not sufficient to obtain a signal from the particles. However, scattering from the sample appears below the critical angle. This occurs because neutron scattering instruments work not with a single wavelength but rather, even for a ‘monochromatic’ instrument, with a distribution around the set wavelength in order to provide sufficient intensity, and there is also a range of incident angles. The shorter wavelengths will penetrate into the sample below the critical angle and can be scattered, while the mean wavelength is still below the critical angle and would be totally reflected. As a result, the penetration depth and consequently the intensity close to the critical angle are smeared depending on the shape of the angle and the wavelength distributions. The longer-wavelength neutrons with lower energies are also, generally, absorbed more in the sample.

In order to determine the intensity that corresponds to scattering from a certain depth of the sample, our model assumes the scattered intensity to be a product of

(*a*) the contrast in scattering length density at each depth of the sample (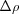) and

(*b*) the intensity profile of the wave, either evanescent or transmitted, at that specific depth.

**Figure 4:**
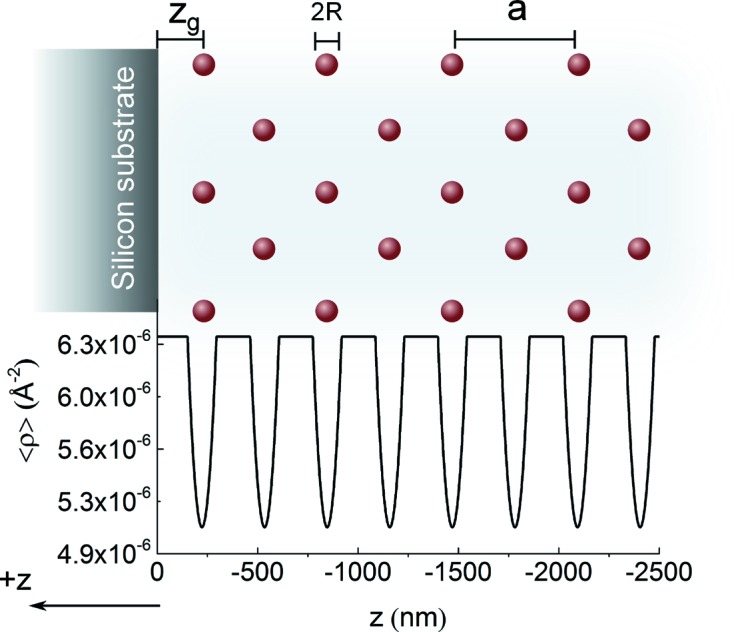
SLD profile for a dispersion of particles with radius *R*, lattice parameter *a*, and a gap between the structure and interface labelled as 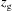, in D_2_O next to a silicon surface. Note that 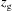 is defined as the distance between the centre of the first layer of particles and the interface.

Knowing the concentration, shape and structure of the particles, the fractional occupancy of the material at each distance from the interface can be calculated. Slicing the sample into planes parallel to the reflecting surface, the scattering length density (SLD) of each plane is then calculated as 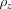 and the SLD contrast as a function of depth *z* is then
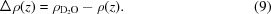
Fig. 4 ▸ shows the SLD profile calculated for 9 wt% PS3 (*R* = 72 nm) latex in D_2_O, *i.e.* lattice parameter *a* = 400 nm which is separated with a gap 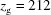
 nm from the solid interface.

The intensity of the evanescent wave at a depth of *z* as a function of wavelength and angle is calculated as
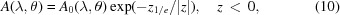
where 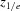 is the penetration depth of the evanescent wave, 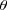 is the angle in the distribution around 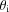 and 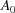
(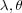) is the initial intensity of the wave. Note that the reason we use the absolute value of *z* is that, in this paper, *z* increases moving away from the reflecting interface towards the crystal and the depths of different parts of the sample are shown with negative values (Fig. 4 ▸).

As described by equation (3), the penetration depth is a function of the incident angle and the critical angle, the attenuation coefficient, and the wavelength of the beam. The different components that contribute to the attenuation coefficient are listed in Table 1 ▸. Values for an X-ray scattering experiment are shown as a comparison in Table 2 ▸. For a neutron experiment, the attenuation coefficient of this system is 1.6 cm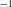, whereas that of an X-ray experiment is more than two orders of magnitude larger, which causes the limited penetration depth of X-rays and increased surface sensitivity of GISAXS.

The model described in this paper accounts for the wavelength spread and the angular divergence of the beam. Both instruments used in this study have the expected triangular function for the distribution of wavelengths from a mechanical velocity selector (Dash & Sommers, 1953 ▸; Hammouda, 1992 ▸). For simplicity in the calculations, a triangular distribution is considered for angular dispersion as well, but these can be changed according to the shape of the resolution functions. By creating a triangular distribution around the wavelength and each incident angle (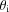), a matrix for probability function *P*(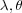) is defined accordingly. The probability function consists of the probabilities for possible angles in columns and possible wavelength in rows.

Angular divergences of 0.065° for D22 and 0.063° for NG3 SANS were calculated, using the following equation: 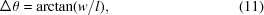
where *w* is the collimation width and *l* is the sample to detector distance of the instrument.

Our code implementing the model creates a three-dimensional matrix for the penetration depth for all incident angles and wavelengths within the triangular distribution around the central value of these quantities. Then the relative scattered intensity for each incident angle is calculated as
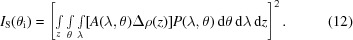
Our calculated intensity comes from an addition of coherent scattering amplitudes. The calculation could readily be altered to allow for a finite longitudinal coherence length that would tend to flatten the decay of intensity with incident angle.

**Figure 5:**
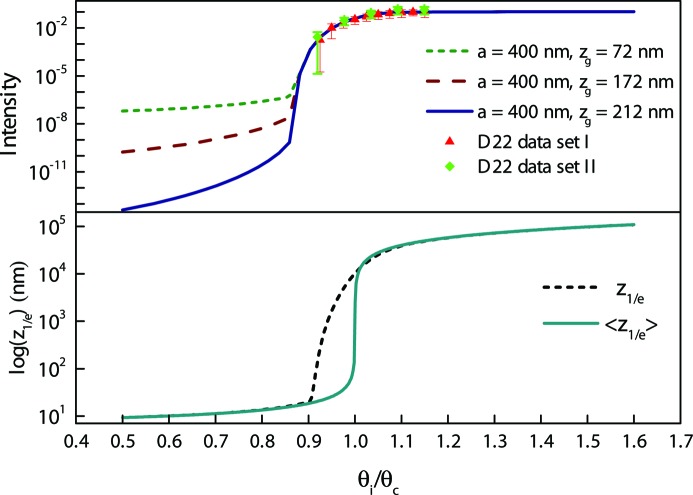
Relative GISANS intensity calculated by the model for PS3 latex with lattice parameter *a* at different separations *z* 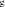 from the interface are shown with lines and the experimental data from two measurements of the same sample are shown as scattered points with error bars (top figure). The corresponding penetration depth at each angle is represented in the bottom plot with (dashed line) and without (solid line) wavelength spread.

In a system where the structure forms with a gap from the interface that is unknown, understanding instrumental artefacts becomes crucial to be able to determine from where in the sample the signal is being recorded. This is usually not a problem in GISAXS experiments, since X-rays do not penetrate deep into the sample and scattering is not recorded from such a structure. The change of the intensity as a function of incident angle, estimated by the model, is shown in Fig. 5 ▸. Intensities are calculated for particles forming a cubic close-packed structure with a (111) face parallel to the solid surface with lattice parameter *a* = 400 nm. The different curves represent the expected scattering intensity when the closest layer of particles is found at distances 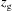 = 72, 172 and 212 nm away from the solid surface. In order to compare the model with experimental data, first-order Bragg peaks on a line at constant 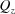 were chosen as regions of interest. These regions of interest are shown as rectangles around the peaks in Fig. 3 ▸. This choice for region of interest allows analysis of the signal dominated by the first layer of particles near the interface and avoids some of the complication of the three-dimensional nature of the structure. Regions of interest were chosen to be in the same position relative to the specular reflection peak and have the same number of detector pixels for each of the incident angles. The regions were chosen to include the whole peak. This allows the program to estimate the shape of the peak and make a model fit to the peak intensity. The Bragg peaks, for both negative and positive 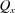 values, at each angle were fitted with both Gaussian and Lorentzian functions, keeping a flat constant background. The mean values for the integral of the Gaussian fit between top and bottom regions of interest are plotted against the model in Fig. 5 ▸. The estimated uncertainties are calculated from the difference between the Gaussian and Lorentzian integrals.

If no instrument effects are included, the intensity as a function of incident angle would have a sharp increase at the critical angle, while depending on the instrument resolution this change is smeared close to the critical angle. This smearing has not been modelled previously. Our model calculates this effect and demonstrates that the changes to the observed intensity are very significant. It provides a good match to the experimental data. The model can be used for other instruments by simply changing the parameters such as the dimensions of the collimation apertures and the wavelength spread that determine the instrument resolution. The model has also been tested for data measured on a different latex sample on the NG3 SANS instrument at NCNR, which uses a more relaxed wavelength resolution than D22. The peak intensities from this sample recorded with NG3 SANS also show results that follow the calculations of the model (Fig. S3 supplementary document). Since the wavelength distribution has a larger FWHM for the NG3 SANS instrument, the smearing of the curves is more pronounced for these measurements.

Some other features that arise from resolution and instrument effects can be identified in the scattering. The refraction of the incident beam produces a streak between the observed direct beam on the detector and the ‘horizon’ that represents the interface between the silicon and the liquid in the sample. Similar streaking/distortion caused by refraction has been discussed previously (Hamilton *et al.*, 1996 ▸; Busch *et al.*, 2011 ▸). This is more marked for measurements with a larger wavelength spread. This wavelength-dependent angular dispersion can also modify the observed shape of the diffraction spots and cause them to be streaked. The alignment of these streaked artefacts will depend on both the spread of the incident beam and the angle of the scattered beam at the interface. These features are indicated in Fig. 2 ▸.

As shown in Fig. 5 ▸, curves with 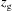 = 72, 172 and 212 nm differ from each other significantly below the critical angle. In that region however, the intensity that would be recorded by the instrument becomes orders of magnitude weaker than that which can be measured readily. This means that even increasing measurement times to hundreds of hours would not provide sufficient data to distinguish these models as the background will restrict the precise determination of the signal. It is, however, useful to consider both what is determined from the present simple experiments and what might be measured for other samples and contrasts that are specifically designed to identify different depths of the sample.

One could design an experiment by adding strongly absorbing materials such as boron which can increase the attenuation coefficient of the sample by two orders of magnitude. The motivation for such an experiment is that the separation of the intensity curves corresponding to different distances from the interface will extend to higher angles and may be measurable. According to our model, for this choice of materials, the relative intensity would decrease by two to three orders of magnitude and even with longer measurement time direct measurement of the separation of the curves for different 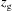 values will be challenging (see Fig. S5 supplementary information). Proposed bright new sources may provide sufficient flux for such an experiment in the future.

## 5. Conclusions   

A model has been developed that explicitly takes into account not just the optical effects of the evanescent wave with absorption but also scattering that may be the dominant influence in many neutron experiments. The penetration depth is calculated accounting for instrument resolution and sample effects. The calculations can be used for any instrument when the resolution function and the structure of the sample are defined. These calculations highlight the difference between experiments with X-rays and with neutrons.

As a test system for our model, the self-assembly of latex particles close to solid/liquid interfaces was studied using GISANS experiments. The particles give rise to scattering from ordered arrays and the calculations show that this arises from depths of up to several micrometres from the interface. The scattering shows a powder-like pattern without a preferred orientation when illuminated with a beam in normal incidence (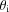 = 90°) that passes through the full depth of 2 mm of the dispersion in the sample holder. These GISANS results indicate that the orientationally ordered structure of the particles is found to depths of several micrometres but they cannot directly determine the thickness of this relatively deep layer. The range of measurable intensity and the instrument resolution do not permit the determination of the structure in regions of just a few nanometres at the interface.

GISANS is a powerful technique to study the structures at solid/liquid interfaces. When illuminating the sample at grazing incidence, structures up to several micrometres can be studied (Fig. S4 supplementary document). Only a small scattering volume is probed directly by the evanescent wave, which is typically less than 10 nm from the interface. This gives weak scattering compared to that observed from the transmitted wave, which can provide signal from distances of 10 µm or more. Local measurement techniques such as QCM-D and ultrasonic thickness measurements can be more effective than neutron scattering for depths of a few nanometres. Highly ordered structures that provide strong diffraction peaks, particularly those that occur at high angles, can be suitable for neutron experiments. Quantitative fitting of the measured intensity always depends on the effects that have been described. All factors that contribute to the attenuation coefficient, which for the case of neutrons may frequently be dominated by scattering rather than absorption, need to be included, and this is an important difference from GISAXS.

There are few widely available programs for quantitative interpretation of GISANS data and these have not been optimized for the type of experiment that has been described in this paper. Improved software is needed to analyse GISANS data for structures that are buried, such as those near solid/liquid interfaces. The present work has provided a simple means to estimate the changes of GISANS intensity as the incident beam angle increases. The calculations have been tested against measurements on model systems with two different sizes of particles that were measured on two different instruments. However the application of the model is not limited to these samples or instruments. Apart from the application in the interpretation of measured data, this approach could be incorporated readily into computer simulation software and used to predict results prior to an experiment, thus improving the design of the experiment by optimizing the sample and instrument settings so as to observe specific features.

## Supplementary Material

Supporting information file. DOI: 10.1107/S1600576717007518/ge5039sup1.pdf

## Figures and Tables

**Table 1:** Contributions of the various compounds of the sample to the absorption of neutrons (Sears, 1992 ▸)

Material	D_2_O	H_2_O	Silicon	Polystyrene
σ_abs_/*V* (cm^−1)^	0.00	0.01	0.00	0.00
σ_coh_/*V* (cm^−1)^	0.51	0.00	0.11	0.03
σ_inc_/*V* (cm^−1)^	0.14	5.37	0.00	3.90
σ_tot_/*V* (cm^−1)^	0.65	5.85	0.11	3.93
SLD/10^−6^ (Å^−2^)	6.35	−0.56	2.07	1.41

**Table 2:** Contributions of the various compounds of the sample to the absorption of X-rays (data correspond to Cu *K*α *E* = ∼8 keV) (Chantler *et al.*, 2001 ▸; Sears, 1992 ▸)

Material	D_2_O	H_2_O	Silicon	Polystyrene
σ_tot_/*V* (cm^−1^)	10.3	10.3	152.8	4.4
SLD_r_/10^−6^ (°^−2^)	9.4	9.4	20.0	9.6
SLD_i_/10^−6^ (Å^−2^)	−0.03	−0.03	−0.45	−0.01
