# Supplementary material for: Grazing-incidence small-angle neutron scattering from structures below an interface
Source: J Appl Crystallogr. 2017 Jul 7;50(Pt 4):1066–74. doi: 10.1107/S1600576717007518 (PMC5541351; doi:10.1107/S1600576717007518)
Supplement: Supplementary file 1 [file j-50-01066-sup1.pdf]

Grazing-incidence small-angle neutron scattering from structures below an interface

Shirin Nouhi, Maja S. Hellsing, Vassilios Kapaklis and Adrian R. Rennie

1. Definition of variables

The table below defines the variables used in the paper:

**Variable**	**Definition**
λ	Wavelength
θ_i	Incident beam angle
θ_f	Outgoing beam's angle with the vertical plane (the angle between the projection of outgoing beam on the horizontal plane and plane-plane intersection)
α	Outgoing beam's angle with the horizontal plane (the angle between the projection of outgoing beam on the vertical plane and plane-plane intersection)
$z_{1/e}$	Penetration depth of evanescent wave (shown as $z_{1/e}$ in some literature)
z	Depth into the sample (negative into the dispersion and positive away from the reflecting surface)
z_g	Spacing of the lattice from the silicon surface
μ	Attenuation coefficient
σ	Scattering cross section
ρ	Scattering length density (SLD)
$\Delta\rho$ (z)	Scattering length density (SLD) contrast in depth z
$A_O(\lambda,\theta)$	Initial intensity of evanescent wave illuminating the sample
$A(\lambda,\theta)$	Intensity of evanescent wave at a certain depth of the sample
$I_S(\theta_i)$	Scattered intensity
a	Lattice parameter
$P(\lambda,\theta)$	Probability function

2. Procedure for cleaning surfaces:

The crystals were cleaned first with dilute Decon 90 for three to four minutes and then rinsed with water. Further cleaning is made by placing the crystal in a glass dish with the polished side facing up. Several drops of sulfuric acid* (98%) are added so as to spread completely on the surface and then approximately the same amount of water is added to acid on the crystal. Adding water to the sulfuric acid causes the acid to heat and boil on the surface. After about five minutes the surface is extensively rinsed with water. Acids cleaning can be repeated up to three times until the surface becomes completely hydrophilic.

*Personal safety equipment has to be used during the cleaning with acid.

3. Wavelength distribution data for the experiments with D22 and NG3-SANS

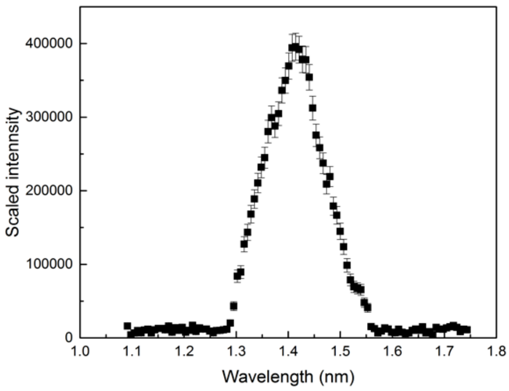

Figure S1. Wavelength spread measured on D22, ILL for λ = 1.4 nm, sample to detector distance 12 m, aperture 20 mm and source to sample distance of 17.6 m.

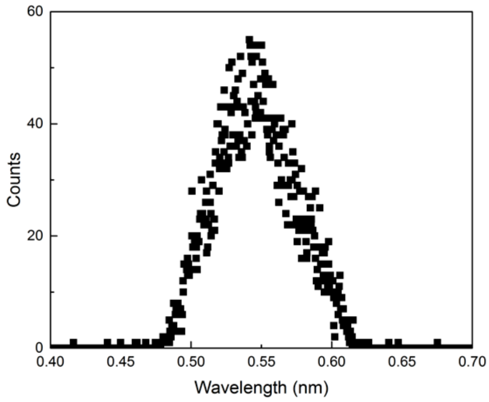

Figure S2. Wavelength spread measured on NG3, NIST for λ = 0.55 nm. The data were provided by Dr J. G. Barker, NCNR for the same velocity selector configuration but with extra neutron guides to provide an adequate count rate.

Figures S1 and S2 show measured wavelength distributions for the neutron instruments used in the experiments that are the expected triangular shape. Triangular functions fitted to these distributions have been used in the model calculations described in the paper.

4. Comparison of data measured at NG3 SANS with calculations

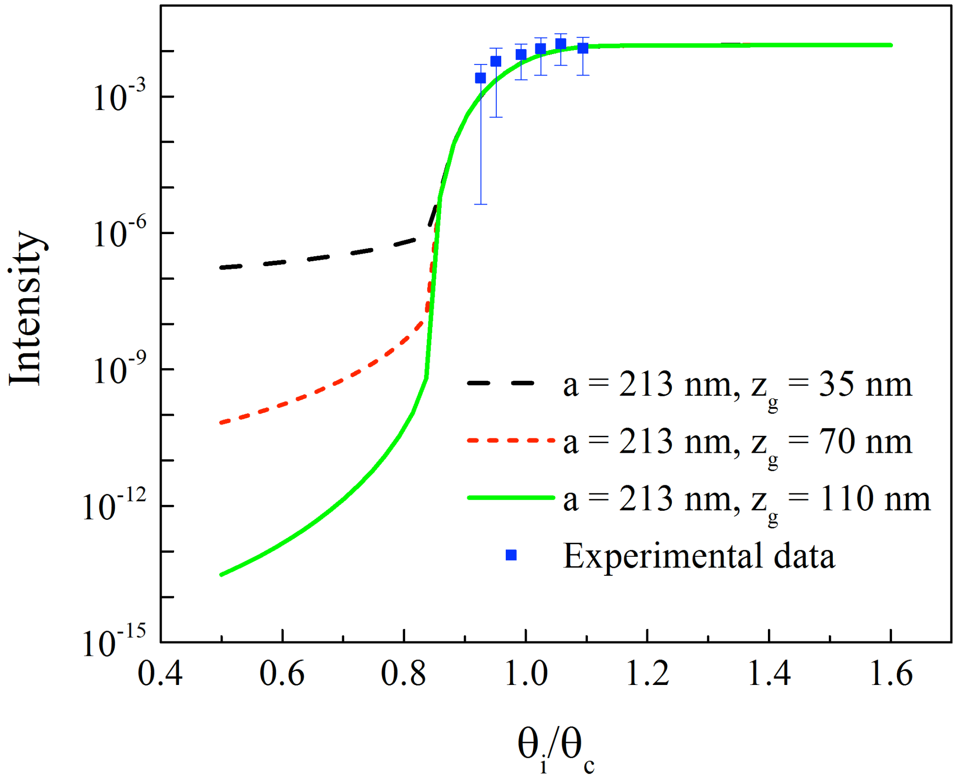

Figure S3. Model calculation of relative intensity and the measured experimental data for diffraction peaks for PS11 particles at Si/D_2O interface measured with NG3 SANS. The model calculates the scattering intensity for λ = 1.2 nm, 12.4% wavelength resolution and 0.063° angular divergence.

5 Comparison of effective penetration depth with and without instrument resolution

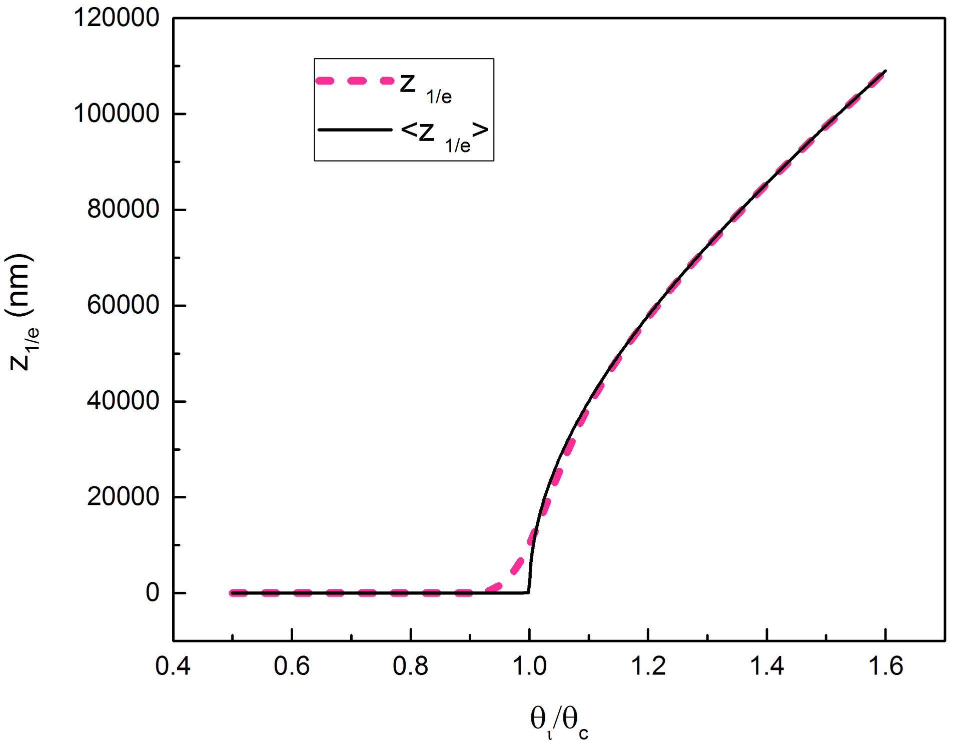

Figure S4. Penetration depth with ($z_{1/e}$) (dashed line) and without the instrument resolution effects of angular divergence and wavelength spread ($<z_{1/e}>$) (solid line) for the instrument configuration used on D22.

6. Possible enhanced surface sensitivity with strongly attenuating samples

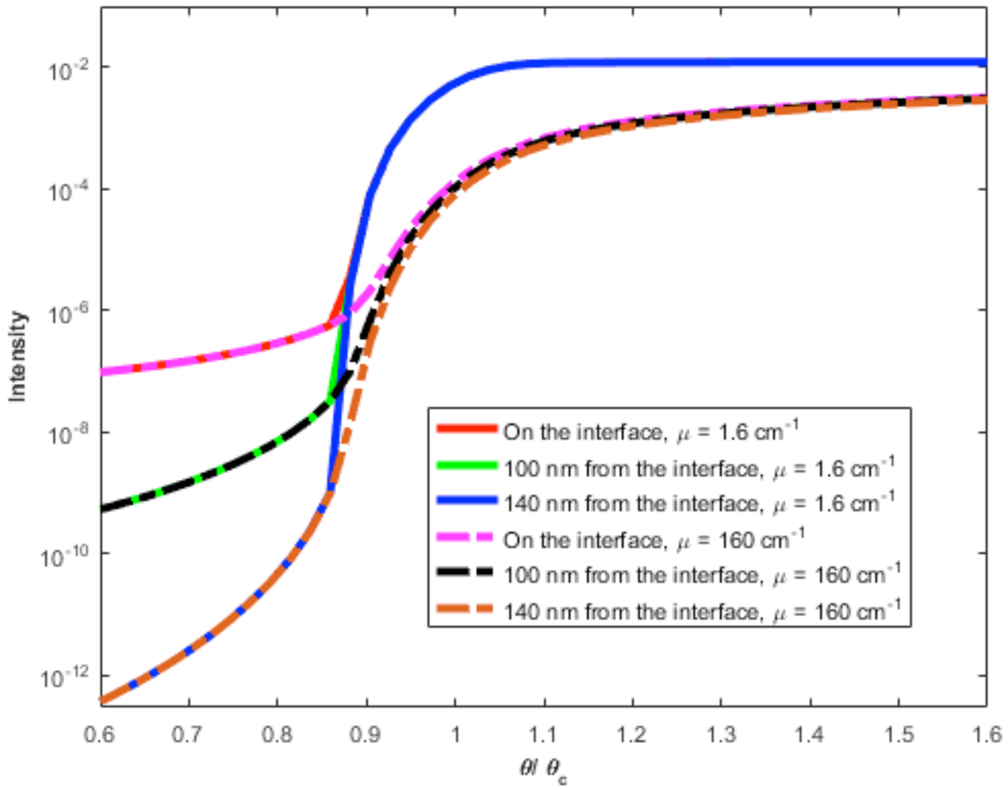

Figure S5. Estimated relative intensities for a sample with an attenuation coefficient 100 times larger than that of the Si/D_2O /polystyrene latex used in the experiments described in the paper. Note that the intensities are shown in an arbitrary scale, this suggests that analysis of shape of the curves can be useful for comparing the differences. In principle much higher sensitivity (few hundred nm) to surface scattering could be obtained with strongly absorbing samples but the choice of materials and stabilizers for colloidal particles would need to be made very carefully. Increasing attenuation by a large factor by means of scattering is not straightforward and would also increase background.

7. Probability function used in the model calculations

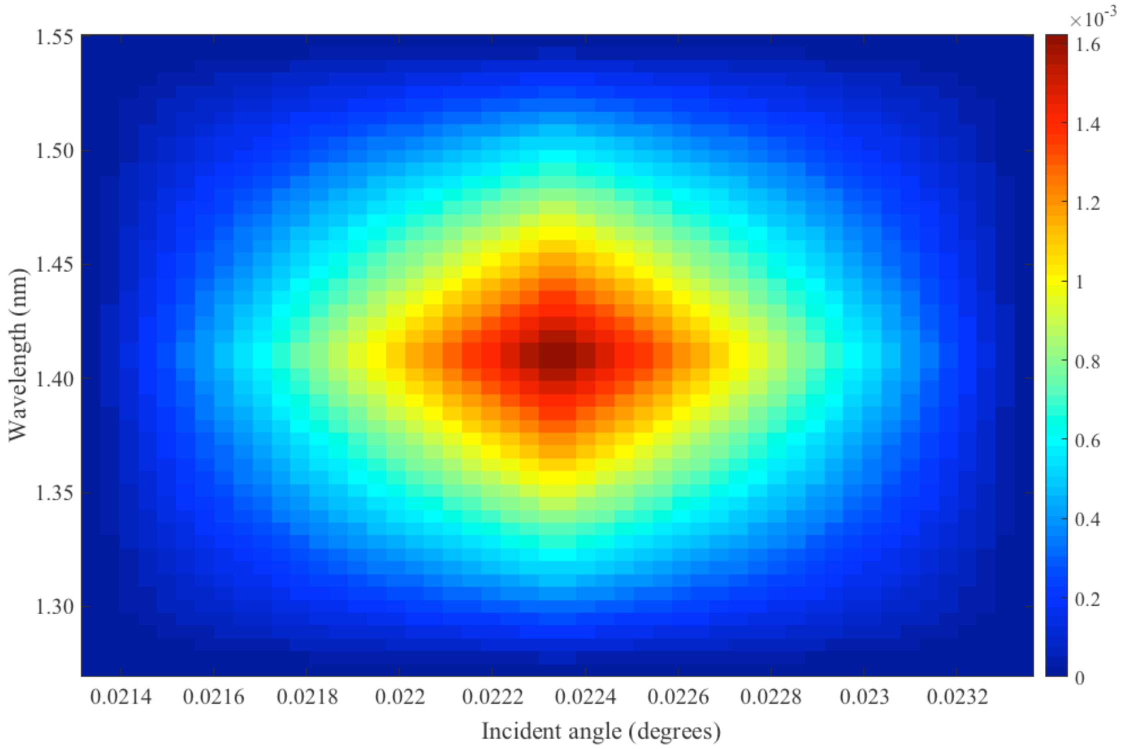

Figure S6. Distribution function for the 1.4 nm wavelength and an example incident angle which in this figure is 0.0223 deg. Sum of the probabilities for all the wavelengths is equal to 1 and same for the angles.

8. Summed intensities for the PS3 latex measured with D22

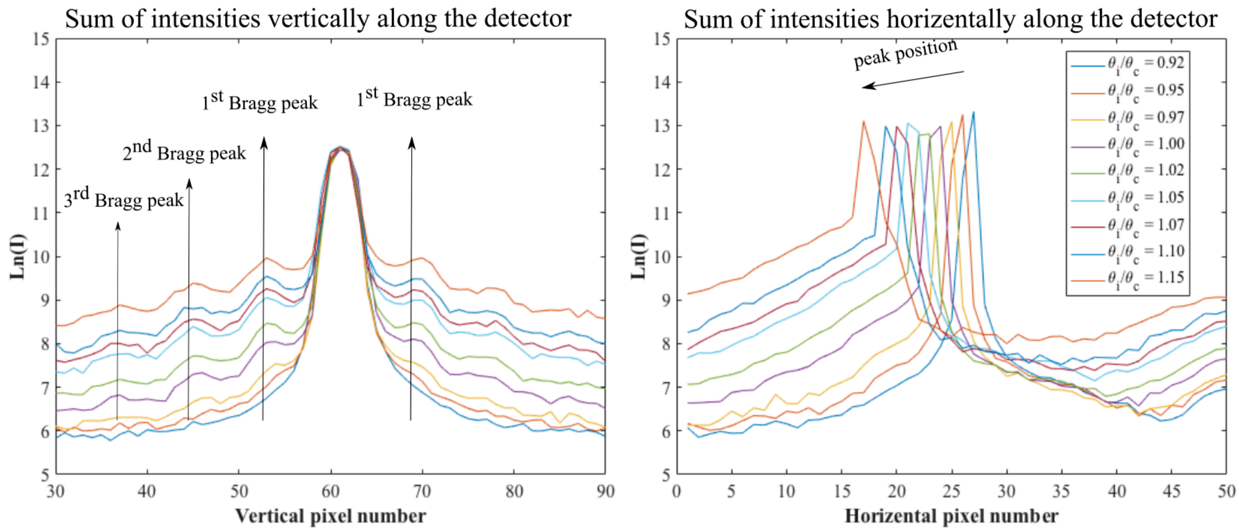

Figure S7. Sum of the intensities along the horizontal (left, in plane scattering) and vertical (right) directions on the detector at different incident angles. The legend indicating angles applies for both plots. The position of the Bragg peaks in the vertical direction (along Q_x) remains constant with incident angle while the horizontal position is shifted together with changes in position of the specular reflection. These plots allow the relative position and width to be assessed.

9. Bare surface analysis

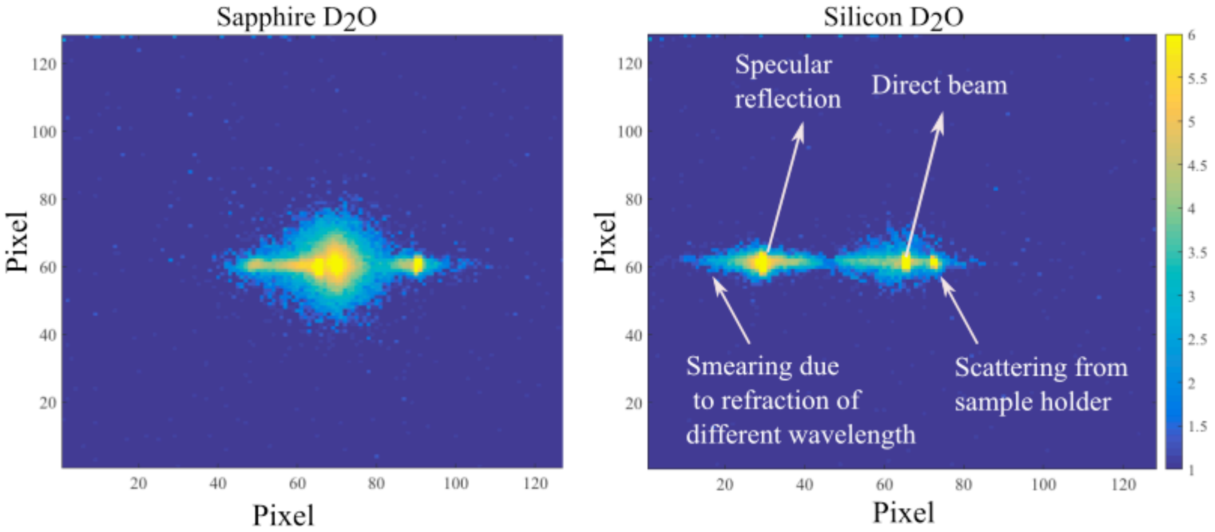

Figure S8. GISANS data for silicon/D_2O at $\theta_i/\theta_c = 0.8$ (right) and sapphire/D_2O at 0.5 degrees (left). The low critical angle for sapphire compared to silicon gives rise to stronger refraction effects and penetration of the beam to the sample holder.
